# Promoting Resilience in the Age of COVID-19 Pandemic: A New Era of Strategic Foresight

**DOI:** 10.14336/AD.2020.1011

**Published:** 2021-02-01

**Authors:** Hanna Lu, Jing Li, Xi Ni

**Affiliations:** ^1^Department of Psychiatry, The Chinese University of Hong Kong, Hong Kong SAR, China; ^2^Department of Sociology, The Chinese University of Hong Kong, Hong Kong SAR, China

Severe acute respiratory syndrome coronavirus 2 (SARS-CoV-2) has caused an ongoing global COVID-19 pandemic [1]. Although Hong Kong has had a good record on controlling the COVID-19 outbreak, a third wave is starting to spread across the whole city. During the past 3 months, the cumulative number of daily infections (3720) accounts for 75.5% of the total number of COVID-19 cases (4926). Among these cases, the median age was 48 years, with nearly 70% cases were young (37.1%) and middle-aged adults (34.8%).

**Figure 1.**
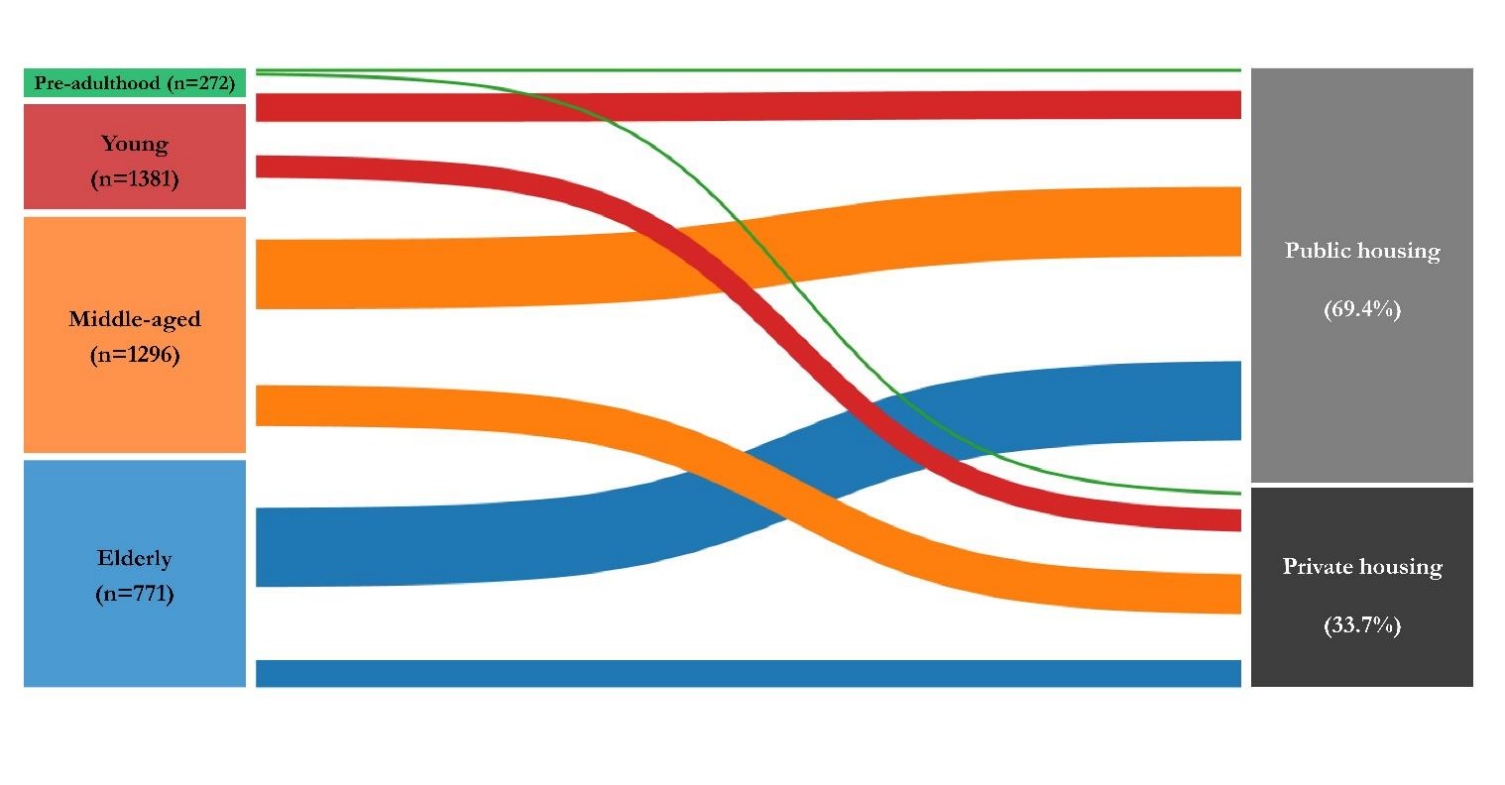
The distribution of confirmed cases featured with housing conditions and age in Hong Kong’s third-wave outbreak of COVID-19.

In the context of the age-specific distribution, however, potential vulnerable individuals may be overlooked behind the third-wave surge. Based on the available residency data (https://chp-dashboard.geodata.gov.hk/covid-19/en.html), we found 69.4% of patients lived in public housing and 33.7% lived in private housing (Fig. 1). When stratified the data by housing conditions and age, we observed that the incidence rates under different housing conditions were similar in children and adolescents (i.e., pre-adulthood) (public: 2.5%; private: 2.7%), increased in young adults (public: 16.9%; private: 11%) and middle-aged adults (public: 28.8%; private: 14%). Notably, there was a striking difference in elderly patients, of which the incidence rate reached 21.2% in public housing, nearly triple that of private housing (6%).

Why housing conditions? The housing conditions in Hong Kong, to a great extent, represent the socioeconomic status (SES) of the individuals who lived in [2]. During the third-wave outbreak, prominent active community transmission has been highlighted in public housing estates. This should be a wake-up call for the government to pay special attention to the individuals with lower SES and old age, because these people may be the real vulnerable ones hidden behind the age-specific distribution.

Another point to be highlighted here is the dynamic relationship between SES, chronological age and resilience. Although the link between SES and clinical outcome has been established [3], SES, as the key component of reserve capacity [4], has a great importance and significance for resilient response, especially among the elderly when they are facing with diseases, unexpected stress and high-level of uncertainty. Resilience represents the capacity to cope with stressful life events across the lifespan [5], which is interrelated with the function of central nervous system (CNS) and psychological conditions. Therefore, promoting and strengthening physical resilience, as well as psychological and mental resilience, by means of individual- or community-based health services or online psychological support could be employed as an adjuvant therapy for coping with the pandemic. For instance, the therapy should be an adaptive management that may involve at least three sequential steps: (1) monitoring the mental status, cardiovascular conditions and sleep quality through smartphones or wearable devices; (2) identifying the disturbances (i.e., warning signs); (3) delivering necessary psychosocial support to the ones who demonstrate warning signs or to the ones with less reserve capacity (i.e., elderly with low SES) in real-time.

Collectively, the key characteristics of the third-wave outbreak highlight the questions and challenges for the Hong Kong government and decision makers. Beside of routine treatment, developing an adaptive management for enhancing the resilient response to COVID-19 pandemic should be integrated into healthcare services, not just in the acute phase, but also in the post-COVID future.
